# Malaria intermittent preventive treatment in Nigeria: a qualitative study to explore barriers

**DOI:** 10.1186/s12879-021-06135-2

**Published:** 2021-05-13

**Authors:** Fatima Mahmud Muhammad, Saharnaz Nedjat, Haniye Sadat Sajadi, Mahboubeh Parsaeian, Abraham Assan, Reza Majdzadeh

**Affiliations:** 1 Department of Epidemiology & Biostatistics, School of Public Health, Tehran University of Medical Sciences, Tehran, Iran; 2 Knowledge Utilization Research Center, University Research and Development Center, Tehran University of Medical Sciences, Tehran, Iran; 3 Global Policy & Advocacy Network (GLOOPLAN), Accra, Ghana; 4 Department of Epidemiology & Biostatistics, School of Public Health, Knowledge Utilization Research Center and Community-Based Participatory-Research-Center, Tehran University of Medical Sciences, Tehran, Iran

**Keywords:** Malaria, Intermittent preventive treatment, Barriers, Pregnant women, Nigeria

## Abstract

**Background:**

While the use of sulphadoxine pyrimethamine (SP) is effective in preventing malaria infection during pregnancy, there are challenges limiting its uptake in Nigeria. This study aimed at exploring the barriers to IPTp usage among pregnant women in Kano state - Nigeria.

**Methods:**

This is a qualitative study. The purposive sampling strategy was used for identification and selection of 14 key informants for interviews. In addition, six focus group discussions (FGDs) were conducted with pregnant women (3 FGDs) and married men (3 FGDs). The conventional content analysis method was used to interpret meaning from the content of the data. MAXQDA 10 software was used for data management and analysis.

**Results:**

Poor policy implementation, poor antenatal care attendance, inadequate access to intermittent preventive treatment at the community levels, lack of sustainable funding, and poor community engagement emerged as major barriers to IPTp use in Nigeria.

**Conclusion:**

While the political will to allocate sufficient financial resources could help improve service delivery and IPTp usage among pregnant women, community participation is critical to sustain the gains.

## Background

Malaria is a major public health challenge in several low-middle income countries (LMICs). Pregnant women are among the most vulnerable groups affected by the disease. In the year 2018, an estimated 11 million pregnant women residing in the sub-Saharan Africa were infected with malaria [1]. Besides, 29% of all pregnancies are at risk of malaria [1]. This is life-threatening as it poses substantial health risk to pregnant mothers, the fetuses and the neonates [2].

However, the good news is that malaria is preventable and curable. Intermittent preventive treatment in pregnancy (IPTp) is given at routine antenatal care regardless of whether the pregnant woman is infected with malaria or not [3] to prevent maternal malaria infection episodes, maternal and fetal anemia, placental parasitaemia, low birth weight and neonatal mortality [4, 5]. As a result, the World Health Organization (WHO) guidelines on antenatal care highlight the need of countries to improve utilization of malaria prevention initiatives [6], including opportunities to expand use of IPTp with sulphadoxine pyrimethamine (SP).

Although great strides have been made over the past two decades to increase IPTp-SP uptake globally, a recent report by the WHO indicates a decline in progress in many African countries, including Nigeria [3]. Moving forward, several efforts have been made by the Government of Nigeria to address the burden of malaria. They include: scale-up activities of intermittent preventive treatment for pregnant women (IPTp); mass media campaign on long-lasting insecticidal nets (LLINs) use, and scale up of malaria case management [7]. Yet, the prevalence of malaria in Nigeria remains high – ranging from 19.7 to 72.0% across states [8, 9]. Pregnant women that have received at least two doses of IPTp is still low [10]. Furthermore, pooled data showed a moderate coverage (of about 18.7%) of IPTp with a wide variation of IPTp usage across Nigeria [10]. According to the recent demographic health survey, about 40.4% pregnant women do use IPTp [11]. This is still low to realize the vision of a malaria free world by 2030.

Quantitative studies have been used to determine the burden of malaria and risk factors influencing use, coverage and access to IPTp in Nigeria [12–14]. Major limitations of these studies include the inability of study design to capture the comprehensiveness of barriers and facilitators of intermittent preventive treatment (IPT) usage among pregnant women. In addition, there are limited evidences in the use of qualitative approach in exploring the phenomenon in Nigeria.

The study aimed at exploring the stakeholders’ perspectives regarding barriers to IPTp usage among pregnant women in Nigeria, through the equity lens. It is anticipated findings could be used to improve IPTp usage among pregnant women in malaria control program.

## Methods

### Study setting

This study was conducted in Kano state, located in North Western Nigeria. It is the second most populous state in the country with an estimated 13.4 million people. The state was selected for the study due its high level of malaria burden compared to other regions [12]. For example, malaria prevalence in the state is approximately 32.4% [11]. In addition, maternal mortality ratio in the region is high, i.e., about 1025 per 100,000 births [15].

### Participants and sampling

We employed snow ball and purposive sampling strategy to select participants for face-to face in-depth interview and Focus Group Discussions (FGDs). Interviews were conducted with 14 malaria stake holders at the national and state level. The maximum variation sampling approach was used to capture responses from varied participants, thereby enhancing the comprehensiveness of our findings [16]. Participants, mainly, policy makers in malaria prevention, health workers, malaria experts and community heads were recruited from three local governments in the states. This includes Nassarawa, Fagge and Kano municipal respectively. Six FGDs were conducted – 3 FGDs were held with pregnant women and 3 FGDs with married men. Each focus group consisted of about 8–10 participants. Data for health workers and pregnant women was gathered across three hospitals – all stationed at different communities. This enabled us to capture wide range of perspective from participants irrespective of their socioeconomic status and geographical location. The selected hospitals were: 1) Aminu Kano Teaching Hospital, 2) Abdullahi Wase Specialist hospital, and 3) Murtala Mohammed Specialist Hospital. Interviews were conducted by FM. The three FGDS for married men and the in-depth interviews for the community heads were conducted in the communities within the three local governments unlike the other interviews conducted in the hospital. Community contact persons assisted in recruiting the eligible participants for the FGDs. The FGDs were conducted in a venue arranged by the community heads (Table 1).

**Table 1:** Characteristics of key informants interviewed

Key informant and FGD	Abbreviations	Number
National malaria director	NMD	1
Regional state malaria coordinator	SMC	1
Malaria experts	ME	6
Health care providers/Matron ANC units	HCP	4
Community heads	CH	2
Pregnant women	PW	3
Husbands	H	3

### Data collection tool and procedures

All interviews were audio recorded. Field notes were also taken where necessary. Separate pre-tested semi- structured interview guides were used for data collection during both the key informant interviews and FGDs. Interview questions covered: challenges of IPTp policy implementation, attendance of women for ANC, IPTp distribution in ANC units, accessibility of IPTp in the communities, knowledge about adverse effects of malaria during pregnancy, facilitators and barriers of IPTp usage. Interviews lasted between 20 and 45 min – when saturation was reached [17].

### Data processing and analysis

Data was transcribed verbatim. The conventional content analysis approach was used to interpret meaning from the content of data due to the inductive nature of this qualitative design [18]. Some of the transcripts were translated from local language to English and verified by experts to enhance accuracy. Transcripts were analyzed by FM and SN using a coding scheme developed from the topics. Data were thoroughly examined by researchers, and relevant perceptions were coded as concepts. Codes related were then summarized to form categories. Next, RM and MP developed the categories, sub-category and the themes. AA, HS and the whole research team ensured that the themes were linked to research questions and study objectives. The themes were compared and discussed to enhance understanding of data. MAXQDA 10 software was used for data management and analysis.

### Quality control and assurance

To enhance the rigor of the study, several researchers independently assigned pre-specified codes to the data [19]. First, five interviews were double-coded by FM. The remaining interviews were double-checked by SN. Next, the entire research team thoroughly discussed the findings for completeness and accuracy. Following that, some data were returned to the interviewees to check the correctness of interpretations. Interviews and data analysis were done at the same time. This enabled us to explore alien responses that emerged during interviews [16].

## Results

A total of 14 face-to face interviews and 6 FGDs were conducted. Stakeholders considered for the interviews included national malaria director, state malaria coordinator, other malaria experts, community members and health care providers. The age of pregnant women ranged from 17 to 40 years. Again, majority of them had attained primary education (50%), secondary education (35%), and about 15% had no formal education. Further, approximately 40% of them were rural dwellers while 60% were from urban areas. The following themes emerged from the analysis: policy implementation; attendance of women for ANC; distribution of IPTp in hospitals; accessibility of IPTp in the communities; and strengthening of IPTp service delivery. Refer Table 2 below.

**Table 2:** Categories, sub-categories and themes

		Categories	Sub-categories	Codes
1	Barriers of Intermittent preventive treatment use	Policy implementation	1–1 Financial obstacles (NMD,SMC,ME)	Inadequate budget for implementation of policies
			1–2 Political obstacles (NMD,ME,HCP)	High population density in endemic areas, corruption in the health system
			1–3 Social obstacles (NMD,ME)	Political reluctance
			1–4 Geographical obstacles (NMD)	Hard to reach areas having rivers and mountains
2		Attendance of women for ANC	2–1 Education (NMD,ME,SMC,HCP)	Low education status of pregnant women
			2–2 Husbands’ Support (ME,HCP,PW)	Some husbands don’t support their wives attending ANC due to cultural believes, low educational status or financial status.
			2–3 Awareness creation (ME,HCP,PW)	Some pregnant women are not aware of the importance of attending ANC including the effect of Malaria in pregnancy
3		Distribution of IPTp in hospitals	3-1Availability of IPTp (HCP,ME,PW,H)	IPTp is little or sometimes unavailable in public hospitals,.
			3–2 Coverage of IPTp (ME,HCP)	Low coverage of IPTp
			3–3 Monitoring of IPTp in ANC wards (NMD)	No proper monitoring to ensure a secure supply of IPTp
4		Accessibility of IPTp in the communities	4–1 Out of pocket payment for IPTp (PW,H,ME,CH)	IPTp is not given for free at PHC
5	Facilitators of Intermittent preventive treatment use	Strengthening IPTp service delivery	5–1 Supervised treatment and providing relevant information to pregnant women (ME,HCP)	Training of health care providers on IPTp, the need to improve the quality of services in health facilities, directly observed therapy should be done in all health facilities as a routine
			4–2 Community involvement (CH,H,ME)	

### Implementation of malaria policies

#### Financial obstacle

Most key informants revealed financial barrier as the major limitation to effective policy implementation targeting IPTp usage. Based on our findings, there is lack of sustainable funding for malaria programs. Governments mainly rely on foreign aids to fight malaria which is not enough, considering the high population growth of the country.

“*We have about 1,200 pregnant women attending the antenatal care monthly in this hospital. In a year, we have nearly up to 16,000. How much does a pack of IPTp cost? Providing three packs for each of these women costs 4.8 million Naira (13,445 Dollars). Hence, providing IPTp for all pregnant women is a huge burden on the government”. – Malaria Expert from Murtala Mohammed Teaching hospital, Kano.*

#### Political obstacle

According to stakeholders, reaching the entire population of Nigeria with malaria interventions require stronger political commitment. However, participants asserted that there is poor political willingness to this cause. Interviewees believed this has contributed to insufficient investments in the provision of IPT in public hospitals. Based on their opinion, political commitment was required to advance progress in the fight against malaria in the country - FGD, a married man.

A malaria program focal person complained that “*…*. *after we finished training the health workers about malaria issues in pregnancy and how to administer IPT, a local government chairman would just come to give another task changing them from the ANC units” - Policy maker at national level.*

### Attendance of women for ANC

#### Poor educational status

Almost all the focal persons interviewed complained of poor attendance of pregnant women during ANC in Kano state compared to other regions of the country. According to stakeholders, turnout was unsatisfactory and fascinated by the poor educational status of women in the locality.

“*About 58% of pregnant women had at least an ANC visit in Kano state. Some pregnant women delayed the visits till their third trimester, so as to have one of the IPTp doses. Besides, it is during such visits that nurses and midwives talked on malaria. Their educational status contributed to their understanding in attending the antenatal care”- Malaria expert at Abdullahi Wase specialist hospital.*

#### Poor male engagements and support for maternal care

Policymakers, experts and pregnant women also lamented the inadequate male engagements and support for maternal care, including: provision of needed financial assistance to support progress of the initiative; ensuring that their wives acquire basic health education and more importantly accompanying them during the ANC so as to be well-informed of their health condition. Views from experts include: *‘The men should help the society by making sure that their wives are educated and financially empowered. Women who are resourceful will always have the financial means to attend ANC.”- Malaria expert at Aminu Kano teaching hospital.*

### Distribution of IPTp in hospitals

#### Inadequate availability of IPTp in healthcare facilities

The findings from almost all the FGDs conducted with pregnant women revealed availability of free IPTp. Informants confirmed that IPTp prescribed for the pregnant women were to be paid for thereby limiting its access and use, especially among the poor who could not afford to pay. “*we were given hematinic as part of the free drugs, but IPTp was not included in the package –* Pregnant woman during FGD. Further, the health professionals also confirmed that “it *has been many years free IPTp and mosquito nets were distributed to pregnant women. - Health care provider.*

### Accessibility of IPTp

#### High out of pocket payments for IPT

Most of the married men interviewed confirmed that they did not buy IPTp for their wives when they were pregnant due to financial constraints. Majority of the married men also stated that their wives only visited the hospitals when it was time for delivery. The informants complained about the cost of health care especially at the primary health care (PHC) units because majority of users at that level still could not pay for the drugs. A community head mentioned that “*we needed the government to provide us with free drugs in the PHC units, especially the IPT, since some couldn’t afford to pay the fees.”- Community opinion leader.*

### Strengthening delivery of IPTp service

#### Poor supervision of treatment

While experts mentioned supervision of treatment as an important step to facilitate the uptake and coverage in the health facility, they lament on the poor supervision on the part of health workers to enhance use of IPTp by their clients. “*The training of nurses and midwives about IPTp should be given much attention because they are the best people to corporate with, and in this situation, directly observed therapy should be done in all health facilities as a routine.”-Policy maker at state level.*

#### Poor community participation

Most participants emphasized on the importance of community involvement. Opinions of experts were that improved community participation is key to a successful delivery of primary health care (PHC). That is, according to interviewees, active engagements of community members can foster effective delivery of malaria programs including usage of IPTp. However, based on our findings, only small group of dedicated community members do promote malaria control. Participants therefore proposed that, *“just as people use the monthly sanitation day to spray insecticide in the community, the same should be done to promote usage of IPTp”* - Community head. This, they believed *“can make the pregnant women, husbands and community members know more about the malaria prevention during pregnancy”.*

## Discussion

The findings of this study provided insight on barriers to IPTp usage.

Four overarching themes emerged explaining the phenomenon. They are: poor policy implementation, absenteeism during ANC visits, inadequate availability (due to increased population size) and financial accessibility (due to limited budget allocation) to enhance the use of IPTp. On the other hand, improved supervision of treatment and community participation emerged as the major facilitators to strengthen service delivery of IPTp. Studies conducted in Uganda and Malawi have revealed similar findings [20, 21]. The study revealed poor ANC attendance by pregnant women as one of the major barriers to IPTp use. Similar studies reported from Ghana and Malawi indicated that irregular and late ANC visits were the key factors for low uptake of IPTp [22, 23] and the reason majority of pregnant women received just a single dose during their period of pregnancy.

Unavailability of SP in ANC units was identified a major challenge to IPTp usage. Quantitative studies conducted in the Southern and Western parts of Nigeria also identified lack of free IPTp thereby limiting its usage [24, 25]. While IPTp is to be provided for free, most of the respondents in the community mentioned that the SP at the PHC units was allegedly sold to patients including the poor who could not afford to pay. This finding was similar to the study reported from Uganda where women were asked to pay for SP whenever it was out of stock [18]. Further, most pregnant women did not s receive the needed financial support from their husbands to enhance usage of IPTp use. Again, several studies have revealed instances where pregnant women were afraid to take SP due to sociocultural barriers [14] All these have renewed the importance of awareness creation of IPTp usage in PHC [26] through active community participation mechanism due to its effectiveness [27].

### Strengths and limitations

The study captured responses from experts with wide range of experiences and background, thereby enhancing the comprehensiveness of our findings. Again, we used various data collection strategies including in-depth interviews, focused group discussions and review of secondary data, which help enhance methodological rigor. Our research team comprises of different researchers with varied backgrounds including epidemiologists, health economist and policy makers. This enabled us to address reflexivity, i.e., individual beliefs, judgements and practices that might have influenced the interpretation of our findings. Still, the study was not without limitation as participants might have had underlining rationale to the responses due to their political and social affiliations. Hence, our findings should be interpreted with caution.

## Conclusion

Malaria infection during pregnancy remains a major public health concern. A call for action to enhance the use of IPTp-SP is relevant and timely. While political commitment is required to make further progress in the uptake of IPTp-SP, improved community participation and sustainable funding mechanism are critical to ensure sustainability of malaria prevention initiatives.

## Data Availability

The data can be made available upon reasonable request from the corresponding author.

## Abbreviations

ANC: Antenatal care
CH: Community head
FGD: Focused group discussion
H: Husband
HCP: Health care provider
IPTp: Intermittent preventive treatment in pregnancy
ME: Malaria experts
NMD: National malaria director
PW: Pregnant women
SMC: State Malaria coordinator
SP: Sulphadoxine pyrimethamine
WHO: World health organization

## References

[1] World Health Organization (2018). World Malaria report.

[2] Ashley EA, Dhorda M, Fairhurst RM, Amaratunga C, Lim P, Suon S, Sreng S, Anderson JM, Mao S, Sam B, Sopha C, Chuor CM, Nguon C, Sovannaroth S, Pukrittayakamee S, Jittamala P, Chotivanich K, Chutasmit K, Suchatsoonthorn C, Runcharoen R, Hien TT, Thuy-Nhien NT, Thanh NV, Phu NH, Htut Y, Han KT, Aye KH, Mokuolu OA, Olaosebikan RR, Folaranmi OO, Mayxay M, Khanthavong M, Hongvanthong B, Newton PN, Onyamboko MA, Fanello CI, Tshefu AK, Mishra N, Valecha N, Phyo AP, Nosten F, Yi P, Tripura R, Borrmann S, Bashraheil M, Peshu J, Faiz MA, Ghose A, Hossain MA, Samad R, Rahman MR, Hasan MM, Islam A, Miotto O, Amato R, MacInnis B, Stalker J, Kwiatkowski DP, Bozdech Z, Jeeyapant A, Cheah PY, Sakulthaew T, Chalk J, Intharabut B, Silamut K, Lee SJ, Vihokhern B, Kunasol C, Imwong M, Tarning J, Taylor WJ, Yeung S, Woodrow CJ, Flegg JA, Das D, Smith J, Venkatesan M, Plowe CV, Stepniewska K, Guerin PJ, Dondorp AM, Day NP, White NJ, Tracking Resistance to Artemisinin Collaboration (TRAC) (2014). Spread of artemisinin resistance in plasmodium falciparum malaria. N Engl J Med.

[3] WHO Reproductive Health Library (2016). WHO recommendation on intermittent preventive treatment of malaria in pregnancy. The WHO reproductive health library.

[4] Wilson NO, Ceesay FK, Obed SA, Adjei AA, Gyasi RK, Rodney P (2011). Intermittent preventive treatment with sulfadoxine-pyrimethamine against malaria and anemia in pregnant women. Am J Trop Med Hygiene.

[5] Anto F, Agongo IH, Asoala V, Awini E, Oduro AR (2019). Intermittent preventive treatment of malaria in pregnancy: assessment of the sulfadoxine-pyrimethamine three-dose policy on birth outcomes in rural northern Ghana. J Trop Med.

[6] Tunçalp Ӧ, Pena-Rosas JP, Lawrie T, Bucagu M, Oladapo OT, Portela A, Metin Gülmezoglu A (2017). WHO recommendations on antenatal care for a positive pregnancy experience-going beyond survival. BJOG..

[7] National Malaria Elimination Programme (NMEP) NPCN, National Bureau of Statistics (NBS), and ICF International (2016). Nigeria malaria indicator survey.

[8] Okwa OO (2003). The status of malaria among pregnant women: a study in Lagos, Nigeria. Afr J Reprod Health.

[9] Adefioye O, Adeyeba O, Hassan W, Oyeniran O (2007). Prevalence of malaria parasite infection among pregnant women in Osogbo, southwest, Nigeria. Am Eur J Sci Res.

[10] Nigeria F (2014). National malaria strategic plan 2014–2020: a road map for malaria control in Nigeria.

[11] National Population Commission - NPC/Nigeria and ICF (2019). Nigeria demographic and health survey 2018.

[12] Dawaki S, Al-Mekhlafi HM, Ithoi I, Ibrahim J, Atroosh WM, Abdulsalam AM (2016). Is Nigeria winning the battle against malaria? Prevalence, risk factors and KAP assessment among Hausa communities in Kano state. Malar J.

[13] Gajida A, Iliyasu Z, Zoakah A. Malaria among antenatal clients attending primary health care facilities in Kano state, Nigeria. Ann Afr Med. 2010;9(3):188–93. 10.4103/1596-3519.68352. 10.4103/1596-3519.68352 20710113

[14] Iliyasu Z, Gajida AU, Galadanci HS, Abubakar IS, Baba AS, Jibo AM, Aliyu MH (2012). Adherence to intermittent preventive treatment for malaria in pregnancy in urban Kano, northern Nigeria. Path Glob Health.

[15] Maternal nachpiKs (2020). Maternal new born and child health program in Kano state.

[16] Assan A, Takian A, Aikins M, Sari AA. Designing and conducting stratified multistage qualitative health service research: a comprehensive insider’s guide: SAGE Publications Ltd; 2020. 10.4135/9781529735987.

[17] Faulkner SL, Trotter SP (2017). Data saturation. The international encyclopedia of communication research methods.

[18] Hsieh H-F, Shannon SE (2005). Three approaches to qualitative content analysis. Qual Health Res.

[19] Ranney ML, Meisel ZF, Choo EK, Garro AC, Sasson C, Morrow GK (2015). Interview-based qualitative research in emergency care part II: data collection, analysis and results reporting. Acad Emerg Med.

[20] Rassi C, Graham K, Mufubenga P, King R, Meier J, Gudoi SS (2016). Assessing supply-side barriers to uptake of intermittent preventive treatment for malaria in pregnancy: a qualitative study and document and record review in two regions of Uganda. Malar J.

[21] Mathanga DP, Bowie C (2007). Malaria control in Malawi: are the poor being served?. Int J Equity Health.

[22] Nkoka O, Chuang T-W, Chen Y-H (2018). Association between timing and number of antenatal care visits on uptake of intermittent preventive treatment for malaria during pregnancy among Malawian women. Malar J.

[23] Ibrahim H, Maya ET, Issah K, Apanga PA, Bachan EG, Noora CL. Factors influencing uptake of intermittent preventive treatment of malaria in pregnancy using sulphadoxine pyrimethamine in Sunyani municipality, Ghana. Pan Afr Med J. 2017;28. 10.11604/pamj.2017.28.122.12611. 10.11604/pamj.2017.28.122.12611 PMC5839217 29515740

[24] Ameh S, Owoaje E, Oyo-Ita A, Kabiru CW, Akpet OE, Etokidem A (2016). Barriers to and determinants of the use of intermittent preventive treatment of malaria in pregnancy in Cross River state, Nigeria: a cross-sectional study. BMC Pregnancy Childbirth.

[25] Amoran OE, Ariba AA, Iyaniwura CA (2012). Determinants of intermittent preventive treatment of malaria during pregnancy (IPTp) utilization in a rural town in Western Nigeria. Reprod Health.

[26] Oppong FB, Gyaase S, Zandoh C, Nettey OEA, Amenga-Etego S, Anane EA, Adda R, Dosoo DK, Owusu-Agyei S, Asante KP (2019). Intermittent preventive treatment of pregnant women in Kintampo area of Ghana with sulphadoxine-pyrimethamine (SP): trends spanning 2011 and 2015. BMJ Open.

[27] Oo WH, Gold L, Moore K, Agius PA, Fowkes FJ (2019). The impact of community-delivered models of malaria control and elimination: a systematic review. Malar J.

